# Controversies in the treatment of postmenopausal osteoporosis: How long to treat with bisphosphonates?

**DOI:** 10.20945/2359-3997000000275

**Published:** 2020-07-17

**Authors:** Francisco Bandeira, Wesdrey Dantas, John P. Bilezikian

**Affiliations:** 1 Hospital Agamenon Magalhães Faculdade de Ciências Médicas Universidade de Pernambuco Recife PE Brasil Divisão de Endocrinologia e Diabetes do Hospital Agamenon Magalhães. Faculdade de Ciências Médicas da Universidade de Pernambuco, Recife, PE, Brasil; 2 Division of Endocrinology College of Physicians and Surgeons Columbia University New York NY USA Division of Endocrinology, College of Physicians and Surgeons, Columbia University, New York, NY, USA

**Keywords:** Osteoporosis, bisphosphonates, drug holiday, atypical femoral fractures

## Abstract

Osteoporosis is a systemic skeletal disease characterized by reduced bone mass and deterioration of bone tissue microarchitecture leading to an increased risk of fractures. Fragility fractures, especially hip fractures, are associated with a significant reduction in the physical function and quality of life of affected patients, as well as increased mortality, leading to a major financial impact on health care. Many drugs have been registered for the treatment of osteoporosis and very recently, a new anabolic agent, romosozumab, has been approved in some countries. Despite the expansion of efficacious antiresorptive and anabolic therapies in recent years, a concomitant increase in concerns have been raised by physicians, patients and the lay press about the potential for adverse events, especially atypical femoral fractures (AFF) following prolonged use of bisphosphonates. Whatever the mechanism(s) may be, direct or indirect, linking prolonged bisphosphonate use to atypical femoral fractures, this adverse event is very rare in comparison to the magnitude of risk reduction of typical osteoporotic fractures. An estimated 162 osteoporosis-related fractures are prevented for each atypical femoral fracture associated with an anti-resorptive medication. Until a risk calculator for predicting risk of atypical fractures, becomes available in clinical practice, and we view this as an unlikely scenario, it is up to the physician to consider continuing or discontinuing bisphosphonate use after the critical 3-5 year period of treatment with zoledronic acid or alendronate, but close monitoring for the residual bone effects overtime should be planned. For other bisphosphonates, in which no residual effects are expected, drug holiday is usually not applied.

## INTRODUCTION

Osteoporosis is a systemic skeletal disease characterized by reduced bone mass and deterioration of bone tissue microarchitecture leading to an increased risk of fractures ( 1 ).

Although the fragility fracture risk is determined by many factors (e.g; fall risk, family history, body mass index, and age), in 1994, the World Health Organization (WHO) developed an operational definition of osteoporosis based on bone mineral density (BMD), as determined by dual energy x-ray absorptiometry (DXA). Osteoporosis is diagnosed when BMD in the spine, femoral neck or total hip is at or below 2.5 standard deviations (T-score) from peak bone mass of young men or women who have reached skeletal maturity (e.g. 25-30 years old) ( 2 ). BMD is a better independent predictor of fracture risk than the serum cholesterol is a predictor of a cardiovascular event ( 3 ).

Fragility fractures, especially hip fractures, are associated with a significant reduction in the physical function and quality of life of affected patients, as well as increased mortality, leading to a major financial impact on health care ( 4 ). Preventing them is therefore the mainstay of osteoporosis management ( 5 - 15 ).

The prevalence of postmenopausal osteoporosis varies worldwide. In Brazil, the prevalence in women ranges from 15 to 33% ( 16 ). In the United States, the overall prevalence is 10.3%, with projections for 2020 and 2030 of 12.3% and 13.6% respectively ( 17 ). This progressive increase in prevalence is due, at least in part, to aging of the population. In this article, we have singled out a specific area for which there is controversy or uncertainty regarding this important public health problem. The area we explore in this article is ‘how long to treat with bisphosphonate?’.

## PROLONGED TREATMENT

Many drugs have been registered for the treatment of osteoporosis ( 5 - 15 ) and very recently a new anabolic agent, romosozumab, has been approved in some countries ( 18 ). Despite the expansion of efficacious antiresorptive and anabolic therapies in recent years, a concomitant increase in concerns have been raised by physicians, patients and the lay press about the potential for adverse events, especially atypical femoral fractures (AFF) following prolonged use of bisphosphonates ( 19 ).

Given the increased risk, albeit small, of atypical femoral fractures even after prolonged use of bisphosphonates, an interruption of use, the so-called ‘Drug Holiday’ has been recommended. The thinking is that when bisphosphonates are stopped, the risk of atypical femoral fractures fall and, in fact, there are data to support this view. On the other hand, stopping therapy may well increase the risk of traditional fragility fractures, including hip fractures. Thus, stopping therapy becomes a two-fold challenge, in which one must balance two kinds of risks both of which are detrimental to the patients skeletal health ( 20 , 21 ). These two concerns are explored in this article.

## ATYPICAL FRACTURES AFTER PROLONGED USE OF BISPHOSPHONATES

According to the American Society for Bone and Mineral Research Task Force, the diagnosis of AFF is made after exclusion of high-trauma fractures, femoral neck or intertrochanteric fractures or pathological and periprosthetic fractures. All the following major criteria are required for the diagnosis: a) location anywhere along the femur from just distal to the lesser trochanter to just proximal to the supracondylar flare; b) association with no or minimal trauma, as a fall from a standing height or less, c) transverse or short oblique configuration; d) noncomminuted complete fractures, extending through both cortices, with or without a medial spike; e) incomplete fractures involving only the lateral cortex ( 22 ).

Minor features may co-exist but are not required for the diagnosis. These include: a) localized periosteal reaction and increased cortical thickness, b) prodromal localized pain; c) delayed healing; d) the presence of associated factors such as vitamin D deficiency, rheumatoid arthritis, hypophosphatasia and the use of glucocorticoids and proton pump inhibitors ( 22 ).

Due to the relatively short duration of bisphosphonate registration trials (3 or 4 years), the first reports of atypical fractures, which are typically associated with prolonged, continuous use, did not surface until experience with these drugs included long term use. To this point, the first reports of atypical femoral fractures began to be published about 10 years after the pivotal alendronate study (FIT) was published ( 23 ).

The effectiveness and safety of the “prolonged use” of bisphosphonates, a term widely used in the literature to refer to a duration of use of more than 5 years for alendronate or 3 years for zoledronic acid, were evaluated in the extension trials of pivotal studies FIT (alendronate) which became the FLEX study, and HORIZON-PFT (zoledronic acid), the HORIZON extension. In FLEX, a significant reduction in the risk of clinical vertebral fractures was shown in patients who continued alendronate for 10 years compared to those who stopped treatment after 5 years (5.3% for placebo and 2.4% for alendronate; RR 0.45; 95% CI 0.24-0.85). There was no difference in cumulative risk of nonvertebral fractures between the two groups, although the study design was not adequate for the assessment of this outcome measure ( 24 ). The HORIZON extension trial, despite showing a significant reduction in the risk of morphometric vertebral fractures in the group continuing zolendronic acid for 6 years in comparison to the group who stopped treatment after 3 years (RR = 0.51, 95% CI 0.26-0.95; p = 0.035), did not show a reduction of nonvertebral or hip fractures. There were no reports of atypical fractures during these extension trials ( 25 ). Relevant to this discussion are the recent studies that have demonstrated an increase in risk of hip fracture among those who have discontinued most bisphosphonate therapies for only 2 years ( 26 ).

The incidence of age-adjusted atypical femur fracture is 1.78/100,000 patients/year, with bisphosphonate exposure from 0.1 to 1.9 years. It increases to 113.1/100,000 patients/year with exposure from 8 to 9.1 years ( 23 ). Although a potential mechanism underlying the development of atypical femoral fractures during prolonged bisphosphonate use is prolonged suppression of bone turnover with subsequent accumulation of microfractures in hypermineralized bone, this possibility remains a hypothesis to be definitely demonstrated. Whatever the mechanism(s) may be, direct or indirect, linking prolonged bisphosphonate use to atypical femoral fractures, this adverse event is very rare in comparison to the magnitude of risk reduction of typical osteoporotic fractures. An estimated 162 osteoporosis-related fractures are prevented for each atypical femoral fracture associated with an anti-resorptive medication ( 20 ).

In a longitudinal evaluation of The SOCS (Southern California Osteoporosis Cohort Study), the risk of atypical fractures after prolonged BP treatment appeared to be greater in women who achieved the highest BMD during treatment. Likewise, those whose bone density was higher at the onset of BP therapy were also at the greatest risk. For those who remained in the osteoporotic range, the risk is even lower with the continuation of treatment beyond 5 years ( 27 ).

The cause-effect relationship between prolonged BP use and atypical femoral fractures continues to be obscure, particularly in the light of current evidence. Abrahamsen and cols. evaluated the effect of alendronate in a cohort study with a case-control design in a Danish population sample (61990 subjects) on hip fracture and subtrochanteric fractures/diaphyseal femur outcomes. The use of alendronate for 10 years was associated with a 30% reduction in hip fracture risk, with no increased risk of atypical femoral fractures. The authors suggest that other risk factors for atypical fractures deserve greater consideration, such as Southeast Asian ethnicity, hip shape and geometry, use of proton pump inhibitors, and history of diabetes mellitus ( 28 ).

## WHY A DRUG HOLIDAY?

This question is a curious one, actually, because osteoporosis is the only chronic disease for which the concept of a drug holiday has gained currency. In other chronic diseases, such as hypertension, hypercholesterolemia, diabetes, and gout, therapy is virtually always continuous. The argument for why the osteoporosis field is dealing with this question cannot be satisfactorily answered by merely stating the bisphosphonates can be associated with serious side effects. Effective therapeutic classes used for these other chronic diseases are also associated, at times, with serious side effects. But no one would reasonably consider stopping these other medications because of these fears. In osteoporosis, however, there seems to be a double standard with an overestimated emphasis placed on rare side effects, like the topic of this article.

Nevertheless, the clinical experience gained after 24 years of bisphosphonate use introduces the need to consider the risk of paradoxically increased atypical femoral fracture due to its use. A seemingly obvious way to reduce the risk of the latter would be to stop using these drugs after a certain period. This finding was evidenced in a Swedish study in which a 70% annual reduction in the risk of atypical fractures following the last dose of bisphosphonate was observed ( 29 ).

## WHEN TO START A DRUG HOLIDAY?

The evidence available to guide decision-making as to the optimal time to discontinue bisphosphonate use is far from definitive. A task force from the American Society for Bone and Mineral Research has reviewed this topic and proposes recommendations similar to those found in the guidelines of the American Association of Clinical Endocrinologists and the Endocrine Society. These entities recommend a Drug Holiday after 5 years of alendronate use or 3 years of zoledronic acid use *if the individual is classified as low risk at the end of these periods* ( 20 , 29 , 30 ). The corollary to this recommendation is *do not stop bisphosphonate therapy if the patient remains at high risk after these periods of time* .

The FLEX trial demonstrated that the number needed to treat (NNT) to prevent a clinical vertebral fracture, comparing alendronate continuation versus placebo after the initial 5 years of use, was lower in the following groups (characteristics present at the beginning of trial extension, after 5 years of alendronate use): T-score in the femoral neck in the absence of vertebral fractures ≤ -2.5 (NNT = 24), T-score in the femoral neck in the presence of vertebral fractures ≤ -2.5 (NNT = 17) and T-score in the femoral neck, in the presence of vertebral fractures, between -2.5 and -2.0 (NNT = 17). In these groups, the risk of typical vertebral fractures is inversely proportional to NNT, and therefore, a greater benefit (than risk of atypical fracture) is expected to maintain alendronate after the initial treatment period when such characteristics are present ( 31 ). Black has recently confirmed this idea by showing from the FLEX trial that those whose FRAX score for a major osteoporotic fracture is > 23% have a greater risk of fracture when the bisphosphonate is stopped ( 32 ). It would seem most reasonable to continue bisphosphonates in those individuals who remain at high risk even after prolonged use. The study by Black proposes an algorithm whereby a value can be given to the actual risk, above which therapy would be continued.

## WHEN TO STOP A DRUG HOLIDAY?

There are no definitive answers to this question. McNabb and cols., based on data from the FLEX trial, suggests that individuals with T-score in the femoral neck that are up to 0.6 above the threshold used for intervention (-2.5 without vertebral fracture or -2.0 with fracture), are likely to need further treatment with bisphosphonates after a Drug Holiday. T-score at femoral neck greater than -1.9 would imply a lower likelihood that alendronate should be resumed during 5 years of a Drug Holiday, and it is unlikely even to benefit from repeat DXA after this period ( 33 ).

Fracture risk during the drug holiday has been evaluated in some studies. A retrospective cohort study evaluated 39,502 women aged 45 years or more with more than 3 years exposure to BP. Subjects with a bisphosphonate holiday for more than 12 months (11,497) were compared to persistent users (17,123; with more than 50% adherence) and nonpersistent users (10,882; with less than 50% adherence) for incident osteoporosis-related fractures. A total of 5,199 osteoporosis-related fractures (including 1515 hip fractures and 2147 vertebral fractures) were observed. The bisphosphonate holiday group was at decreased risk for osteoporosis-related fractures in comparison to the non-persistent users (HR 0.71; 95% CI, 0.65 to 0.79), vertebral fractures (HR 0.68; 95% CI, 0.59 to 0.78), and hip fractures (HR 0.59; 95% CI, 0.50 to 0.70). Women who had a holiday for more than 12 months, after taking BP for more than 3 years, did not show increased risk for osteoporosis-related fragility fracture, hip, or vertebral fractures compared to current BP users ( 34 ).

In contrast, as stated before, another retrospective cohort in postmenopausal women with more than 80% adherence to BP use for more than 3 years showed 30% increases in the risk of hip fractures when drug holiday exceeded 2 years ( 26 ).

**Figure 1:**
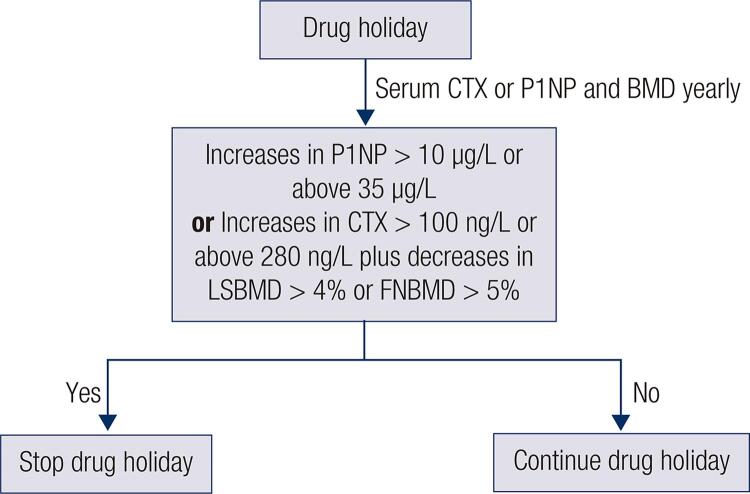
Interpretation of bone density and bone markers changes during drug holiday.

Bone turnover markers (BTM) may also play a role in deciding when to stop a drug Holiday ( Figure 1 ) ( 35 ). The bone formation marker serum N-terminal propeptide of type I collagen (P1NP) and the resorption marker serum C-terminal telopeptide (CTX) have been used in clinical practice to monitor adherence to pharmacological treatment of osteoporosis, as well as to evaluate the efficacy of these drugs. Because they change earlier than BMD, they enable follow-up consultations in shorter time frames, with a view to reducing treatment dropout rates or early identification of suboptimal response.

Protocols recommending the use of BTM during treatment of osteoporosis have been suggested ( 36 ). This algorithm consists of the measurement of P1NP or CTX before starting the anti-resorptive drug and 6 months after, permitting reassessment of the patient’s response and/or compliance. However, it is known that CTX decreases earlier than P1NP, allowing even shorter monitoring period (at 3 months)^.^ Non-significant changes in the serial measurements of these markers would imply the need to strengthen treatment adherence, consideration of secondary causes of osteoporosis, or change in pharmacological class or the route of administration of the drug chosen as the first option.

The usefulness of these markers during a Drug Holiday has been less well studied, but a possible strategy would be to indicate that bisphosphonates should be resumed if serum P1NP is increased to above 35 μg/L or a positive variation of P1NP above 10 µg/L. The same applies to serum CTX, with respective increases to above 280 ng/L or an absolute increase of 100 ng/L, especially in the setting of significant decreases in BMD ( 36 , 37 ) ( Figure 1 ).

In conclusion, until a risk calculator for predicting risk of atypical fractures becomes available in clinical practice, and we view this as an unlikely scenario, it is up to the physician to consider continuing or discontinuing bisphosphonate use after the critical 3-5 year period of treatment with zoledronic acid or alendronate, but close monitoring for the residual bone effects overtime should be planned. For other bisphosphonates, in which no residual effects are expected, drug holiday is usually not applied.
